# Hospital and Institutional News

**Published:** 1916-09-23

**Authors:** 


### HOSPITAL AND INSTITUTIONAL NEWS.

### PRINCE ALBERT'S ILLNESS.

The news of H.R.H. Prince Albert's illness, communicated on September 17, the day after his name had been published in the list of those commended for good service in the Battle of Jutland, is certain to excite the sympathy of the whole nation and a distinct measure of anxiety as well; it is to be hoped that the latter may be allayed by subsequent bulletins. The announcement of September 17 states that the Prince has been invalided owing to an acute abdominal trouble, and that an abscess has been evacuated. To medical ears this bulletin conveys an impression of a distinctly serious illness, though it is good news to hear that the operation has been successfully performed, evidently by Sir Frederick Treves. When it is recalled that Prince Albert's appendix was removed in September 1914, and that it was nine months after that before he could return to duty owing to an unnamed "gastric disorder," it will be realised what responsibilities rest upon his medical, and especially upon his surgical, advisers. Both to their Majesties and to Prince Albert the nation as a whole offers respectful sympathy at the moment, and the best wishes for a happy, speedy, and permanent recovery.

### QUEEN MARY'S HOSPITAL FOR THE EAST END.

A great honour has recently been conferred upon the West Ham and Eastern General Hospital, Stratford, E. Her Majesty the Queen has become patroness of the institution, and has graciously consented to allow the hospital to be re-named "Queen Mary's Hospital for the East End." This hospital serves not only West Ham (itself the seventh largest town in England), but also the populous districts of Leyton, Leytonstone, Wanstead, etc., with a total population of about 900,000. The gracious and kindly action of Her Majesty in allowing her name to be associated with the work will encourage the local workers as nothing else could have done. It will be remembered that on the 29th of last month their Majesties the King and Queen paid a visit to this hospital, when they were received by the President, her Grace the Duchess of Marlborough; the Chairman (Mr. C. E. Leo Lyle), Dr. P. T. S. Nicoll, the Matron (Miss E. A. Sordy), the Secretary (Mr. A. W. Scrivener), and conducted round the institution. Their Majesties were greatly interested in hearing the experiences of the wounded, including seventeen Anzacs, who had come direct from the fighting round about Pozières. At present there is a deficit of about £7,000, but the authorities are undismayed, and it is hoped that Her Majesty's action will result in others interesting themselves in this most deserving charity in order that it may not be necessary to curtail the work. It may be noted that a schoolboy of East Ham, George Sturton, has been made a life governor of this hospital in recognition of his successful collections for its funds: clearly the authorities are alive to the importance of encouraging everyone who can and will help them.

### THE LATE SIR T. LAUDER BRUNTON.

The death of Sir Thomas Lauder Brunton, Bart., M.D., F.R.S., removes not merely a distinguished London physician, but one of the earliest of those who applied experimental laboratory research and findings to clinical medicine. Born in 1844 and educated at Edinburgh, he graduated M.B. there in 1866, and then spent some years in study at Continental universities before settling in London in 1870. He was soon appointed assistant physician to St. Bartholomew's Hospital, which he served for many years in that capacity, as physician and lecturer, and finally as consulting physician. He early laid the foundations of a scientific reputation by his work on pharmacology, then a much-neglected and scarcely recognised science. His work on the Nizam of Hyderabad's Chloroform Commission, though much disputed and controverted, was a piece of research which attracted perhaps more attention than any other of his numerous contributions to medical science, except that on the nitrites. As a practising physician Sir Lauder built up a very large practice, though it cannot be said that his reputation in the medical profession as a clinician was ever as great as that as a research worker and a pharmacologist. He was a prolific writer, received honorary degrees from British and foreign universities, and had been Vice-President of the Royal Society. He is succeeded in the baronetcy, conferred upon him in 1908, by his eldest son; the second son, who was brought up to his father's profession, was killed while serving as a lieutenant in the R.A.M.C. last year.

### THE AMERICAN RED CROSS AND THE WAR.

An interesting report has been issued relating to the assistance rendered by the American Red Cross to the belligerent Powers. Between September 7, 1914, and July 1, 1916, this Society made 217 shipments to the Allied Powers. These shipments consisted of 32,605 packages, and included purchased supplies valued at approximately £73,200 and donated supplies, designated, amounting to £81,780, and undesignated, amounting to £45,400—a total of £200,380. During the same period forty-eight shipments were made to the Central Powers, consisting of 6,667 packages, of which the purchased supplies were valued at £14,350; the designated donated supplies were valued at £38,300, and the undesignated donated supplies at £9,490, making a total value of £62,140. In addition to these shipments, supplies valued at £22,175 were purchased by the American Red Cross and forwarded to France in three shipments. Fifty complete hospital tents were also purchased for the French Red Cross at a cost of £1,759, for which the American Red Cross received reimbursement. In addition to the listed supplies sent to the Central Powers, there were shipped by the American Red Cross to the Austrian Red Cross in Vienna thirty-four cases of antitoxin, valued at £26,395, and to the German Red Cross at Berlin thirty-four cases, valued at £45,915, which were paid for by the Austrian and German Red Cross Societies respectively. The total value of all the goods supplied is thus well over £358,000, and of course the work is still going on.

### THE FIRE AT WREST HOUSE.

A disastrous fire occurred last week at Wrest House, between Luton and Bedford, where some two hundred wounded were under care. Fortunately the alarm was sounded before the fire had spread far; and, thanks to the self-possession of the patients and the devotion of the nurses and orderlies, all the soldiers, thirty of whom were "cot cases," were safely removed from the building. This house, formerly one of the seats of the late Lord Cowper, was given by his nephew, Lord Lucas, to the nation last April for the purpose of a hospital. In it Lord Cowper housed a very fine collection of art treasures, all of which, it is pleasing to hear, have been saved. The extent of the damage done by fire and water is considerable, but it is hoped that it can be repaired. It is stated that the prompt arrival of the village fire brigades from Shefford, Ampthill, and Woburn Abbey, together with the excellent fire drill of the Wrest House hospital staff, alone saved the mansion from total destruction. Motor and horse vehicles conveyed the wounded to Woburn Abbey, Ampthill Park, and Woburn Cottage Hospital. The cause of the fire is unknown at present.

### A RUN OF LEGACIES.

As is the case in other events, legacies to hospitals tend to average themselves out as a source of income, and the smaller hospitals which cannot always depend upon this enviable regularity sometimes benefit by a run of legacies which is exceedingly useful. A present instance of the kind occurs in connection with Worthing Hospital, which has received somewhere about £6,000 in the last seven years from legacies alone. During this time, however, the ordinary income of the hospital has equally regularly shown a deficit, and the curious result arrived at is, notwithstanding the utilisation of part of these legacies to meet the annual deficiency, the capital sum at the hospital's disposal has tended to increase rather than diminish. To count on a continuance of such good fortune would be bad management, and therefore we hope that the public will try to increase the annual subscription list.

### THE NEW HOSPITAL AT MUSWELL HILL.

The gift, and partial endowment by the donor, of a hospital is always a noteworthy event, and a remarkable instance of it has occurred with the purchase, by an anonymous donor, of a big house in Alexandra Park Road, Muswell Hill, which has been extended and equipped as a military hospital. The new hospital possesses accommodation for fifty beds, and after the war is over the intention is to use it for ordinary hospital purposes. It is understood that some six thousand pounds have been spent in converting the building, and that the donor has expressed his willingness to contribute £1,000 a year towards the maintenance of the hospital so long as the war continues.

### EXTENSIONS AT BUXTON.

The Duke of Devonshire recently opened a new wing and new baths which have been added to the Devonshire Hospital at Buxton. Presiding at the hospital's annual meeting on the same day, the Duke made reference to the long connection of his family with the institution, mentioning that it was exactly a hundred years ago that his predecessor, the sixth Duke, occupied that chair. During the year, he said, they had treated 1,100 soldiers, in addition to over 3,000 ordinary patients. The extensions had cost a good sum, but he hoped their indebtedness of £11,000 would be wiped off.

### A NEW CONVALESCENT HOME FOR MERCHANT SEAMEN.

It is most satisfactory to learn that at last the list of war charities includes an important institution for the benefit of the men of the mercantile marine. This has come about through the generosity of Mr. Henry Radcliffe, who has purchased the Passmore Edwards Convalescent Home, Limpsfield, Surrey, with the object of converting it into a convalescent home for seamen of the British mercantile marine. Mr. J. Havelock Wilson, president of the Seamen and Firemen's Trade Union, has been invited to assist in starting the home. The value and heroism of the merchant seamen in this war, whether pursuing their ordinary calling with nothing but their mother-wit to aid them against attacks by submarines, or in the dangerous and devoted task of mine-sweeping, or in the ranks of the Navy itself, have been incalculable, and at the same time very little recognised or even realised by the public. It is therefore a timely thought of Mr. Radcliffe to provide them with a home in which these often homeless men can spend their convalescence after the disease or accident to which their calling may subject them at any time. The Limpsfield Home was originally opened by King Edward VII.

### A NEW ADDITION TO KING EDWARD VII.'S HOSPITAL, CARDIFF.

A gratifying announcement has been made by Colonel Bruce Vaughan at a meeting of the board of management of King Edward VII.'s Hospital, Cardiff. After stating that the maternity wards, which were made possible by Mrs. Nixon's generosity, have been started, he went on to say that a maternity flat for fifty beds would be provided and ready for occupation in January next. The institution will include not only these beds, but the site for a further extension; two labour wards, three baby and ordinary bathrooms, a receiving room with bathroom, and rooms for patients' clothes, together with accommodation for a matron, two labour-ward sisters, two day sisters, one night sister, and four staff nurses. When it is added that an out-patient department will be provided, the value of the maternity flat to the hospital when it seeks as a hospital with a medical school attached to comply with the regulations laid down by examining bodies is obvious. Another stride therefore is being taken towards the goal which the friends of King Edward VII.'s Hospital have in view.

### A HOSPITAL'S JUBILEE.

The Reigate and Redhill Hospital has just commemorated its jubilee, and on looking through the summary of its history from the date of its inception a record of gradual growth and useful work is seen. The institution opened on September 1, 1866, in Nutley Lane, Reigate, with six beds; was removed five years later to its present site, and opened with accommodation for eight patients; 1875 saw a west wing added with an additional eight beds, which number was subsequently increased in 1899 by a further enlargement of the new wing, bringing the total accommodation to twenty-seven beds and three cots; while in 1908 a children's ward was opened, the hospital thus built containing now forty beds.

### V.A.D. ENERGY IN BIRMINGHAM.

Moor Green House, lent by Sir John Holder, is being utilised as an extension of the Highbury (Birmingham) V.A.D. Hospital, and its financial responsibilities are guaranteed by Messrs. Kynoch's workmen, who are also maintaining Highbury. The accommodation is for fifty beds, and the staff consists of two medical officers, a matron, the commandant, and members of the Worcestershire V.A.D. 82, assisted by members of the other V.A.D.s and friends. Extensions have also been carried out at Harborne Hall V.A.D. Hospital (assisted by Messrs. Avery's workmen) for thirty additional beds by the erection of a large open-air ward. Further additions have also been made at Lordswood, Norlands, and Sutton Coldfield V.A.D. Hospitals. The total number of beds provided by the Birmingham (St. John Ambulance Brigade) V.A.D.s is now over 520.

### HOMES FOR THE DISABLED IN LANCASHIRE.

A special committee of the East Lancashire branch of the British Red Cross Society, under the chairmanship of the Lord Mayor of Manchester, has started to raise £100,000 for the acquisition, equipment, and maintenance of a home or homes in Lancashire for totally or permanently disabled sailors and soldiers. The homes will be established under the East Lancashire branch of the British Red Cross Society working in co-operation with the local committees, which have been formed under the Naval and Military War Pensions Acts. The idea is for the institutions to provide the special care needed by those whose private homes, even with the total disablement and supplemental pensions provided by the State, are inadequate for this purpose. It is also felt that such a home or homes should be in a locality accessible to the friends and relations of the patients. Therefore the first home it is hoped to establish will be in the neighbourhood of Manchester.

### A CURIOUS IMPROVEMENT AT CUMBERLAND INFIRMARY.

Among the improvements which have lately been carried out at Cumberland Infirmary is one which, though small in itself, is worth attention, for it is not only suggestive but the result of a Coroner's recommendation. This improvement consists in making the exits from the infirmary more secure, so that, should a patient be inclined to escape, he would have great difficulty in doing so. Hospitals in the past have been somewhat unappreciative of Coroners' recommendations, and the barring of the infirmary's exits is a curious example of acquiescence in a Coroner's ideas. It suggests, too, the old days when a waiting list meant rather those who were anxious to get out than those impatient to get in. The only hospitals from which patients wish to escape nowadays are isolation hospitals or asylums, in which the treatment is generally prolonged.

### THE NURSING OF MALE DEFECTIVES.

Dr. R. Dods Brown, physician superintendent of Murray's Royal Asylum, Perth, in a report provides further evidence on the debated question of the employment of female nurses in the male wards of mental institutions. Owing to the fact that sixteen attendants have joined the Army, a number of female nurses has been engaged. These, of course, have had to undertake the work formerly performed by the attendants, and Dr. Brown declares that the female nursing of male patients has proved satisfactory. Since such nursing is performed under supervision, and there seems little or no difficulty offered by the nurses who are considered suitable to undertake the work, why is it that the advocates of the system are mainly residents North of the Tweed, while the opponents of the female nursing of male patients in asylums are mostly confined to the Southern kingdom? The circumstances created by the war will at least bring the question to the test of experience on a larger scale than ever before.

### MALINGERING AND SELF-MUTILATION.

The experience of all other countries where conscription is an established institution is that a certain small percentage of reluctant recruits will go to the length of self-mutilation in order to produce military unfitness for service. In war-time and in the world-peril of the moment this state of mind is almost inconceivable, and that of those who for profit aid and abet the malingerer or the mutilator is even more anti-social and degenerate. Still, the authorities ought to have foreseen that these problems would be certain to arise, and their recently issued regulations under the Defence of the Realm Act are distinctly belated. As they stand the regulations seem adequate, and it is to be hoped that they will be put in force rigorously and without favour. It is especially noteworthy that it is equally an offence to assist an intending malingerer by producing in him any mutilation or by supplying him with drugs wherewith to "dope" himself. If any addition to the regulations is needed, it is perhaps a proviso that the signing of any certificate of medical unfitness to enable a recruit to impose on a medical board or a Tribunal should be a criminal offence. There is only too much reason to believe that some medical men have been far too lavish in the distribution of such certificates, including even a few with high reputations in the profession for clinical eminence; and that much of the outcry in the gutter press against the vagaries of medical boards is to be traced to such certificates and to the distrust with which all civilians' certificates have come, in consequence, to be regarded.

### TRAINING IN SOCIAL SCIENCE AND ADMINISTRATION.

The expansion of Social Service during the last few years has created a large demand for professional workers capable of taking paid posts of many different kinds under municipal and other civil services, as well as under the now numerous private societies and agencies engaged in some branch or another of charitable effort towards similar ends. Although as yet these posts are not confined to those who have undergone any specified courses of university or other training (either practical or theoretic), there is a distinct and accelerating tendency visible to give preference to candidates who can produce some such evidence of genuine effort to fit themselves for this work. The London School of Economics and Political Science (Clare Market, Portugal Street, W.C.), which is a constituent part of the University of London, has prepared courses of instruction, with syllabuses of lectures and practical work, for those who desire to submit themselves for the University Diploma in Sociology and Social Science. For those whose ambition does not extend quite so far there is the certificate granted by the London School to those who have carried out satisfactorily a prescribed course of less extensive training; and for those voluntary workers who do not aim at becoming salaried officials there is a part-time course of an abbreviated nature along the same lines. The prospectus of all these courses and of various bursaries and exhibitions in connection with them is full of interest; institutionally, perhaps the most important lecture is that which will be given upon "Hospital Almoners" by Miss A. E. Cummins, lady almoner to St. Thomas's Hospital and to the Northcote Trust.

### THE ENCOURAGEMENT OF WOMEN MEDICAL STUDENTS.

An interesting offer has been made by a Pontypridd lady, Mrs. Hopkins Morgan, who is anxious to see more young women from that town making an endeavour to enter the medical profession. Feeling that some, though they may possess the ability to win a maintenance scholarship at the Welsh University, may yet be debarred from becoming lady doctors by their lack of means, Mrs. Hopkins Morgan has offered to pay the three years' maintenance fees, which work out at 63 guineas, for the first Pontypridd woman who secures a medical scholarship. This is a course which might be followed with national advantage by other persons seeking new outlets for their benevolence.

### THE MAN OF DESTINY.

One of the most respected old patients of a large hospital frequently sends a report of his affairs to the institution, and, as is not unusual, seems to take pride in the series of misfortunes which have happened to him. The following is a fairly comprehensive list, which begins with a reference to previous letters. We reproduce it as it stands:—"most of my axidents i have sent you little finger split down the middle ball of thumb cut off reaping—top joint of thumb gone stiff—forried [forehead] cut open with cane [crane] at work right arme cut off at the ebbow with light engin doctors took it of twice after 3 times in one houre & the frost took it right fut twiste half round—left instiff run over at steel works dog bit me throw the caff of leg nose broken when a young man left leg fractured below the nee left thy [thigh] fractured at work right thy fractured at home leg gave way right leg gave way at the infirmery [infirmary] & hurt the gides up the side of leg hed split open coming out of a butchers shop in — street chopped my nee very badley when a boy i have been nearley drounded 5 or 6 times when a boy learning to swim i have nearly been shot 6 times i got nocked down with the first horce at plow & all three horses & plow went over me & did not hurt me saved a throw express traine & passingers from beeing smashed to attams [atoms] at bedford saved 2 ladies from falling down a deap sewerage at narbrough mr curneedy [Carnegie] ought to now of all these i ought to get rewarded by some one to be shore [sure]." Such a chapter of accidents could happen only to a man of destiny, and few of us perhaps would care for a life so much crowded with incident as this.

### RACE SUICIDE ENCOURAGED AT LOUTH.

Institutions all over the country are just now experiencing such difficulty in filling even the humbler staff vacancies that it may be found necessary ere long not only considerably to increase the salaries offered, but also to relax the regulations appertaining to the posts. The Faversham Guardians, in seeking—and seeking successfully—for the conditional exemption of one of the workhouse officials the other day, told the Tribunal that repeated advertisements of the position only brought one reply, and the applicant asked for the impossible salary of £500 a year, or over five times the normal remuneration for the work. At Louth the porter and portress of the workhouse have resigned, "owing to increase of family and no provision for such," their letter adding, "It is certainly a mistake to advertise for young people between twenty-five and forty, no children allowed and none wanted." Worse than a mistake; it is a serious crime against the State to aid and abet race suicide in the way that many corporate employers of labour do. Hospital authorities and Boards of Guardians ought to set a good example over this serious and important question.

### LETTERKENNY AND OMAGH GUARDIANS DEFIANT.

Both at Letterkenny and at Omagh the Guardians are still resolutely bent on thwarting the efforts of the Irish Local Government Board to prevent the appointment of medical officers who are of military age. The Board has treated both these local authorities with patience and lenience in the matter; and has even now, after exhibitions of open defiance and ostentatious disloyalty from both these parishes, only got as far as announcing the intention of surcharging the salaries to the Guardians who vote for the appointments. At Omagh this threat is derided, and the principal spokesman of the Guardians—a Justice of the Peace, by the way—declares in favour of contesting such a surcharge by legal proceedings. The fact is plain enough to anyone who reads the accounts of the debates at the Guardians' meetings that hostility to Great Britain and pro-Germanism are mainly accountable for the truculent attitude of these local bodies. Alike for the sake of the Allied cause and for the future of order and good government of Ireland, it is incumbent on the Irish Local Government Board to take a very firm line and not to deviate in the least therefrom. In the meanwhile the two young doctors concerned are branding themselves with infamy in the eyes of every man who believes the cause of the Allies to be more just than that of the Central Powers.

### A BOGUS WAR CHARITY.

At a meeting of the Central Committee of the Hull and East Riding Voluntary Aid Society, at which it was decided to amalgamate, for the collection of funds under the War Charities Act, the East Yorkshire war charities, the Sheriff of Hull gave a remarkable instance of the necessity for registration. He stated that he had recently received a beautifully printed circular, embellished with imposing photographs, which invited subscriptions for "Rest Homes for Soldiers." He noticed, however, that no committee was mentioned, and that there was merely an address to which subscriptions should be sent. On communicating with the authorities he discovered that the only persons responsible for the publication of this appeal were two bookmakers. The appeal, he added, had brought in several thousand pounds. On reading an instance of this kind we are tempted to wonder whether those who subscribed so carelessly to the appeal are not equally guilty with its promoters. Their irresponsibility, at all events, is an incitement to fraud and an encouragement to rascality.

### THE RATING OF AN ABERDEEN HOSPITAL.

At the recent session of the Aberdeen Burgh Valuation Appeal Court the trustees of the Royal Hospital for Sick Children were successful in obtaining a reduction of the assessment on the old hospital premises and nurses' home from a total of £300 to one of £185. It seems that when the hospital and the nurses were moved last year to new premises the old buildings were taken possession of by the military, and rent was paid to the trustees for just over a year. The authorities have now surrendered the premises, which are unlet and likely to remain so, it is said. The net rental during the time of the military occupation was £285. It seems that the rating authorities have been unusually kind to the trustees in granting this considerable reduction. We have every sympathy with hospital boards in attempts to get their ratings reduced; indeed, we support the exemption of hospitals altogether. But as this latter principle is not accepted by the Government, obviously local authorities are within their rights in fixing substantial valuations. Logically, we cannot quite see why a hospital building should be rated lower when disused than when in use; rather it ought to be the other way round. For the trustees, in this case, will not be called upon for rates until they find a tenant, and when that happens presumably the tenant will not be a voluntary hospital. However, with the efforts of boards to get their rates reduced we sympathise strongly; and offer congratulations to the Aberdeen Royal Hospital for Sick Children accordingly.

### A BOLD SCHEME OF REFORM.

Dr. W. Angus, medical officer of Leeds, is concerned regarding the dirty conditions in which many people of the poorer classes are content to live, and in his annual report he ventures a hint of sanitary compulsion. His suggestion is that "there should be established in each district colonies or compounds under municipal control, under the permanent supervision of sanitary police. When dirty tenants are ejected from houses they should be compelled to live in one of these colonies, where the supervision would be so close that they would be compelled to live a clean life and keep their houses in clean condition. Only when their habits were sufficiently improved would they be allowed freedom to go and live where they liked. The conditions of life would be greatly improved for the children, and the possible stigma less harmful than the alternative of squalor and dirt." We are afraid the idea, though in accord with common sense, is too Utopian to stand a chance of adoption. Dr. Angus's plan is interesting, however, and most people will agree with his declaration that such folk "are a curse to every town, and an important factor in retarding the sanitary progress of the people, because they contaminate their neighbours wherever they go."

### THE SYMPATHIES OF CORONERS' JURIES.

Every now and then a Coroner's jury allows its verdict to be dedicated by the heart rather than the head; decisions such as that recently brought in by a Stafford jury form powerful arguments for the abolition of the jury altogether at Coroners' inquests. Briefly, a waggoner was kicked on the jaw four years ago. The local practitioner thought the jaw was fractured, but the surgeons at the infirmary at Stafford decided against that diagnosis. The man got well and went back to work. In June this year he was again kicked by a horse, this time in the side. In August he developed a swelling of the face, which turned out to be an abscess, and the man died of septic poisoning early in September. A *post-mortem* examination showed that there was no fracture of the jaw, and that the abscess had arisen probably from septic teeth. The jury found, however, that the abscess was caused by the first accident four years ago, and was accelerated by the second accident. Obviously enough this was an attempt by the jury to provide a basis upon which the relatives of the deceased might found a claim for compensation from the employers, and in order to do this they disregarded their oaths and decided against the weight of evidence. Had they known that their verdict will not have the legal effect intended, even in the remotest degree, they might perhaps have retained their integrity and based their verdict upon the evidence.

### THE ORGANISATION OF HOSPITAL SUNDAY PARADES.

Members of the various friendly societies and others in the districts served by Addenbrooke's Hospital, Cambridge, have been trying to do all they can to increase the annual income. To make their annual hospital parade collections a greater success the Secretary-Superintendent of Addenbrooke's has attended a number of them and given addresses on the work and needs of the hospital. Addenbrooke's has benefited to the extent of about £180 over and above the amount received from the same source last year. At Welney, a village of 900 inhabitants, £45 was raised for Addenbrooke's and two other institutions. There is reason to believe that if more of such collections could be organised Addenbrooke's would have a large part of the additional £2,000 a year required to carry on its work. What can be done in Cambridge for Addenbrooke's can be achieved in other places too.

### THE SALARIES OF HEALTH VISITORS.

Notwithstanding the war's effect upon progressive organisation in public-health work and other fields, the recent expansion of maternity and child-welfare schemes and the appointment of trained health visitors continue. The training of the health visitor is becoming increasingly technical, with the result that the salary must rise so as to attract a class of woman able and willing to meet the new standard required. In so far, however, as these appointments are extra-institutional, the salary as a rule is still too low. For instance, Lambeth has lately appointed as health visitor for the southern division of the borough a lady with the following qualifications: She has had sixteen months' experience as a health sanitary inspector at Bradford, previous to which she had been for five years nursing at Scarborough Hospital, where she was eighteen months charge nurse. After being presented with a maternity training scholarship of £25, and taking a full course at the Jessop Maternity Hospital, Sheffield, she obtained the certificate of the Central Midwives Board. The Lambeth appointment as health visitor carries with it a salary beginning at £102 12s. per annum, which rises by £3 a year to £128 5s., with an annual allowance of £10 for uniform. As such salaries go, this may be reasonably good, but can it be considered an adequate reward for some ten years of work and training?

### AN IRISH DOCTOR'S BRAVERY.

A remarkable instance of bravery is reported from Ireland, where Dr. Cornelius C. Hickey, medical officer of Kilkee, lately rescued two persons from drowning, a lady who was being carried out to sea while swimming, and her would-be rescuer, who himself was quickly in difficulties, and had already sunk twice before Dr. Hickey, after bringing the lady to shore, turned back and at last succeeded in extricating him. When it is added that the man and woman who thus owed their lives to Dr. Hickey were themselves described as powerful swimmers, the courage and skill of their rescuer is easily seen. We understand that Dr. Hickey has previously rescued other persons from drowning, and since he possesses the Royal Humane Society's certificate in vellum for life-saving, he clearly deserves not only the gratitude of his neighbours, but whatever further distinction it is in the power of the Society or the authorities to bestow.

### THIS WEEK'S DRUG MARKET.

A slightly healthier tone is noticeable in the drug market, but the volume of business continues small. The expected advance in the price of English refined camphor has now been announced, the advance being at the rate of about 6 per cent.; makers appear to be busy. There is a good demand for potassium bromide, and for prompt delivery some purchasers have been willing to pay a price slightly above makers' quotations. Phenacetin is still dearer, and only small lots are available. Resorcin is practically unobtainable. Salicylic acid and sodium salicylate are neglected, buyers being unwilling to make purchases while the market is so unsettled. English foxglove-leaves continue scarce and rather higher prices are quoted; plentiful supplies have been collected by amateurs, but unfortunately a large proportion of the leaves have not been properly dried, and are, therefore, unsuitable for pharmaceutical use. Cocaine is slightly lower in price. Chamomiles are much dearer in consequence of shortage. Quinine is again rather lower in price. Oil of peppermint is dearer. Cod-liver oil is in very small demand, and prices show no signs of receding; on the contrary, quotations for the best qualities have a tendency to advance.

### TO OUR READERS.

**Contributions are specially invited from any of our readers to these columns. They should deal with topical subjects and news. They must be authenticated for the information of the Editor only. The minimum payment if published is 5s. There is no hard-and-fast rule as to space, but notes of about twenty lines in length are preferred.**

